# A dataset of proteins associated with *Trypanosoma cruzi LYT1* mRNAs

**DOI:** 10.1016/j.dib.2022.107953

**Published:** 2022-02-15

**Authors:** Elizabeth Ruiz Márvez, César Augusto Ramírez Segura, José María Requena, Concepción J. Puerta

**Affiliations:** a Departamento de Microbiología, Facultad de Ciencias, Grupo de Investigación en Enfermedades Infecciosas, Pontificia Universidad Javeriana, Carrera 7 # 40- 62, Bogotá, Colombia; b Departamento de Biología Molecular, Grupo Regulación de la Expresión Génica en Leishmania del Centro de Biología Molecular Severo Ochoa (CSIC-UAM), Universidad Autónoma de Madrid, Madrid 28049, Spain

**Keywords:** Alternative trans-splicing, Interactome, *LYT1 gene*, Pull-down, RNA-binding proteins, *Trypanosoma cruzi*

## Abstract

Post-transcriptional gene regulation in *Trypanosoma cruzi*, the etiological agent of Chagas disease, plays a critical role in ensuring that the parasite successfully completes its life cycle in both of its obligate hosts: insect vector and mammals. This regulation is basically governed by RNA binding proteins (RBPs) through their interactions with *cis*-elements located in the UTRs of their mRNA targets. LYT1 gene, coding for a virulence factor of *T. cruzi*, is expressed into two isoforms: kLYT1 and mLYT1, which play different functions according to their cellular location and parasite life-cycle stages. Whereas kLYT1 exhibits a regulatory role during the epimastigote-to-metacyclic trypomastigote stage transition, mLYT1 acts as a pore-forming protein, relevant for host cell invasion and parasite intracellular survival. Considering the LYT1 biological relevance and the fact that this is a protein exclusive of *T. cruzi*, the protein and its mechanisms regulating the alternative gene expression products are promising targets for therapeutic intervention. In this work, an experimental approach consisting of pull-downs assays followed by proteomic analyzes was carried out to identify the proteins interacting with the different LYT1 mRNAs. The dataset presented here was obtained through three biological replicates using all the different UTRs characterized in the LYT1 mRNAs (i.e., 5´UTR kLYT1, 5´UTR mLYT1, and I and II-type 3´UTRs) as baits, and protein extracts from epimastigotes and trypomastigotes of the 058 PUJ (DTU I) strain. Bound proteins were analyzed by liquid chromatography coupled to mass spectrometry (LC/MS). As a control of non-specificity, the same protein extracts were incubated with *Leishmania braziliensis* rRNA and the bound proteins also identified by LC/MS. In all, 1,557 proteins were identified, 313 of them were found in at least two replicates and 18 proteins were exclusively associated with the LYT1 baits. Of these, six proteins have motifs related to RNA binding, and seven remain annotated as hypothetical proteins. Remarkably, three of these hypothetical proteins also contain nucleic acid binding motifs. This knowledge, beside expanding the known *T. cruzi* proteome, gains insight into putative regulatory proteins responsible for alternative LYT1 mRNAs processing. Raw mass spectrometry data are available via MassIVE proteome Xchange with identifier PXD027371.

## Specifications Table

**Table:** 

Subject	Biology, Molecular Biology (General)
Specific subject area	*Trypanosoma cruzi* proteomics
Type of data	Figures Supplementary tables in Excel file Proteome raw data
How data were acquired	Protein samples were isolated with streptavidin-coated magnetic beads (Promega, Inc., Madison, WI, USA). Magnetic beads with proteins bound to RNA baits were subjected to 3 washes with 50 mM ammonium bicarbonate (1 mL) and digested with trypsin (1 µg) (Promega, Madison, WI, USA) for 5 h at 37 °C, then desalted on a stage tip (C18) and vacuum dried before MS injection for Liquid chromatography-mass spectrometry (LC-MS) analysis. Peptide samples were separated by LC-MS/MS on an Ekspert NanoLC425 (Eksigent) coupled to a 5600+ mass spectrometer (AB Sciex, Framingham, MA, USA) equipped with a nanoelectrospray ion source. MGF peak list files were created using Protein Pilot version 5.0 software (Sciex), and then searched using Mascot (Matrix Science, London, UK; version 2.5.1) [1] on the TAX_Trypanosoma cruzi_5693database (55,107 entries) assuming the digestion with trypsin. Visualization of data was done using the Scaffold program version 4.8.5 (Proteome Software, Inc. Portland, OR, USA).
Data format	The data are raw, filtered and analyzed.
Parameters for data collection	Liquid Chomatography (LC)/Mass spectrometry (MS) analysis of proteins from *T. cruzi* captured by pull-down based on their binding to the UTRs of LYT1 mRNAs.
Description of data collection	Protein extracts from either *T. cruzi* epimastigotes or trypomastigotes were incubated with the 5´UTR kLYT1, 5´UTR mLYT1, 3´UTR-I and 3´UTR-II biotinylated-RNA baits. As a control of non-specificity, the same protein extracts were incubated with *Leishmania braziliensis* rRNA. Afterwards, RNA-protein complexes were retained on Streptavidin-coated magnetic beads (Promega, Inc., Madison, WI, EUA). Samples (three replicates of each pull-down assay per bait) were submitted to trypsin digestion and LC/MS analysis for protein identification. Supplementary Figs. 1 and 2 showed the baits used in each assay.
Data source location	Proteomics Facility Institution: proteomics platform of the CHÚ de Québec (http://www.crchudequebec.ulaval.ca/) Laval University Research Center City: Québec Country: Canada
Data accessibility	Repository name: Center for Computational Mass Spectrometry. Mass Spectrometry Interactive Virtual Environment MassIVE. Data identification number: [doi:10.25345/C54C29]. Code: PXD027371. *Trypanosoma cruzi* proteins identified in pull-down assays aimed to characterize RNA proteins interacting with LYT1 mRNAs ID=8fac68aa1011440089dbfbf7ba1fb488 Direct link to the dataset:https://massive.ucsd.edu/ProteoSAFe/dataset.jsp?task=8fac68aa1011440089dbfbf7ba1fb488 Supplementary materials with the article Repository name: Mendeley Data Data identification number DOI:10.17632/mfxcr5v2dc.1 Direct link to the dataset: https://data.mendeley.com/datasets/mfxcr5v2dc/1

## Value of the Data

• The reported dataset of proteins provides to the scientific community valuable information on proteins involved in the parasite gene expression regulation; in particular, these proteins are potential regulators of LYT1 gene expression, in both epimastigote and trypomastigote stages of *T. cruzi.*
• This dataset could be useful for researchers focus on the discovery of new RNA interacting proteins as well as in the establishment of the protein-protein or protein-RNA interaction networks that regulate gene expression in trypanosomes.
• The new proteins recorded in this dataset, either in the infectious stage (trypomastigote) as in the replicative one (epimastigote) of *T. cruzi*, expand the knowledge of the parasite´s proteome.
• This dataset could also may shed light on *T. cruzi* protein expression patterns according to the parasite stage.

## 1 Data Description

**Fig. 1:**
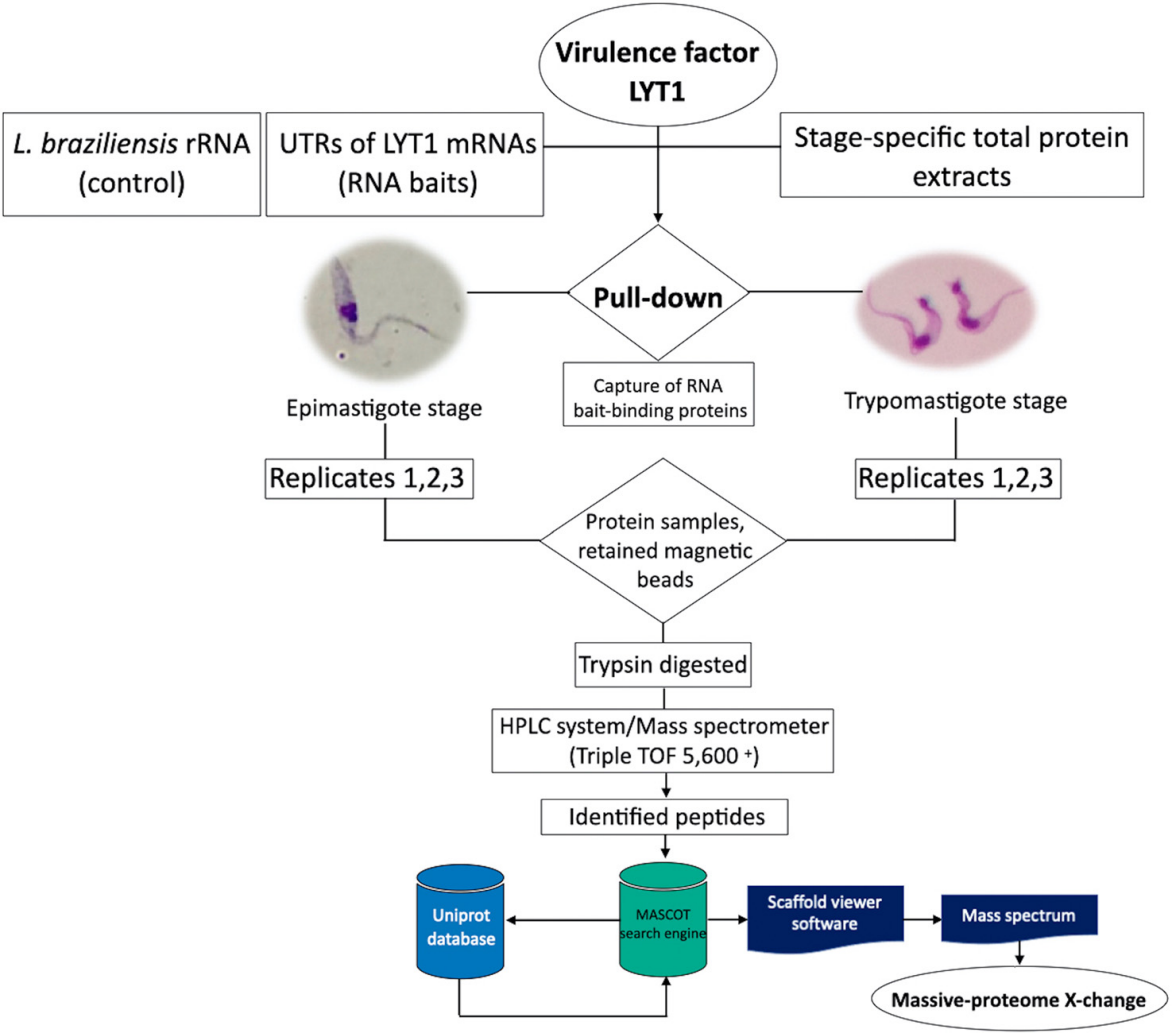
Experimental design aimed to the identification by LC/MS of proteins interacting with LYT1 mRNAs UTRs.

Three independent pull-down assays were performed using all four different LYT1 mRNAs UTRs (5′UTR kLYT1, 5′UTR mLYT1, and type-I and type-II 3′UTRs) as baits. L. *braziliensis* rRNA was used as non-specific RNA bait (control). The captured proteins were analyzed by LC/MS. The entire pipeline is illustrated in Fig. 1.

For each individual experiment, the proteins identified per individual replicate with each bait in epimastigotes and trypomastigotes are listed in the Supplementary Tables S1 and S2, respectively. In all, 628 proteins were identified in the epimastigote samples and 929 proteins in the trypomastigote ones (Excel files contain different sheets for each assay and in turn, each assay includes its own control bait). Supplementary figs. 1 and 2 show the baits used in each replicate and the number given to the samples at the CHÚ de Québec proteomics Service-Laval University Research Center (Québec, Canada). The supplemental Tables S3 and S4, derived from Tables S1 and S2, list the 313 proteins detected in at least two replicates among the different LYT1 mRNA baits, including the L. *braziliensis* rRNA control bait.

As specific LYT1 mRNA-interacting proteins were considered those present in at least two replicates and absent in the control assays. In this manner, eight proteins were recorded among the identified in the epimastigote stage (Table 1) and ten proteins were recorded in the trypomastigote stage (Table 2).

**Table 1:** Epimastigote proteins specifically interacting with the LYT1 mRNAs UTRs.

*T. cruzi* stage	Protein ID	Name protein	UTR bait	Domain	Function	Refs.
Epimastigote	MOQ_004095; TCDM_02013	TcUBP1	*5´UTR mLYT1*	RRM	Regulates mRNA stability	[2]
	TcSylvio_000964	Hypothetical	*5´UTR kLYT1*	G,N,M	Possible GTPase/SRP54	[3]
	TcCLB.510773.20	Vacuolar pyrophosphatase type I	*3´UTR-I*	**–**	Regulates pyrophosphate levels	
	TcCLB.503815.10	Alkyldihydro-xyacetone phosphate synthase	*3´UTR-I*	**–**	Phospholipid biosynthesis	
	TcSylvio_003557	Hypothetical	*3´UTR-II*	dTMP kinase	Thymidylate kinase. DNA synthesis	[4]
	TCDM_10537	Hypothetical	*3´UTR-II*	Pleckstrin (PH) like	Phosphatidylino-sitol binding	[5]
	P06660.1.	Heat shock protein 85 kDa	*3´UTR-II*	TPR	Regulates protein folding	[6]
	MOQ_004172	T complex protein 1	*3´UTR-II*	TCP-1	Regulates protein folding	[4]

**Table 2:** Trypomastigote proteins specifically interacting with the LYT1 mRNAs UTRs.

*T. cruzi* stage	Protein ID	Name protein	UTR bait	Domain	Function	Refs.
Trypomastigote	MOQ_002367	S4	*5´UTR mLYT1*	S4	Structural constituent of ribosome	[4]
	MOQ_000235	Hypothetical	*5´UTR mLYT1*	LRR	–	
	TCDM_14447	TcZc3h10 Hypothetical	*5´UTR mLYT1*	Beta helix	Nucleic acid binding	[5]
	TcSylvio_009788	Hypothetical	*5´UTR kLYT1*	LRR	–	[4]
	TcCLB.508475.10	S20	*5´UTR kLYT1*	S10	Modulates 40S assembly and rRNA processing	
	TcSylvio_000995	Prohibitin	*5´UTR kLYT1*	PHB	Mitochondrial regulation	
	TcSylvio_009078	Hypothetical	*5´UTR mLYT1 /kLYT1*	ForkHead box (FHA)	Transcription factor/DNA repair	
	TcCLB.509793.30	KAP4	*5´UTR mLYT1 /kLYT1*	HMG box	kDNA binding protein	[7]
	TcSylvio_009289	Tol-T	*5´UTR mLYT1/5UTR kLYT1/ 3´UTR-I*	–	Flagellar protein trypomastigote	[8]
	TcCLB.508461.490	L23	*3´UTR-II*	L14/L23	Structural constituent of ribosome	[9]

According to the RNA bait, 7 proteins were identified by their binding to the 5′UTR kLYT1 (1 in epimastigotes and 6 in trypomastigotes), another 7 because their interaction with the 5′UTR mLYT1 (1 in epimastigotes and 6 in trypomastigotes), 3 bound to the 3′UTR I (2 in epimastigotes and 1 in trypomastigotes) and 5 associated with the 3′UTR-II (4 in epimastigotes and 1 in trypomastigotes) (Tables 1 and 2). In addition, three of these proteins were found associated with more than 1 bait: KAP4 (TcCLB.509793.30) and a hypothetical protein (TcSylvio_009078) were identified by their interaction with both 5´UTRs (mLYT1 and kLYT1), and Tol-T (TcSylvio_009289) that interacted with both 5´UTRs and the 3´UTR-I baits.

**Fig. 2:**
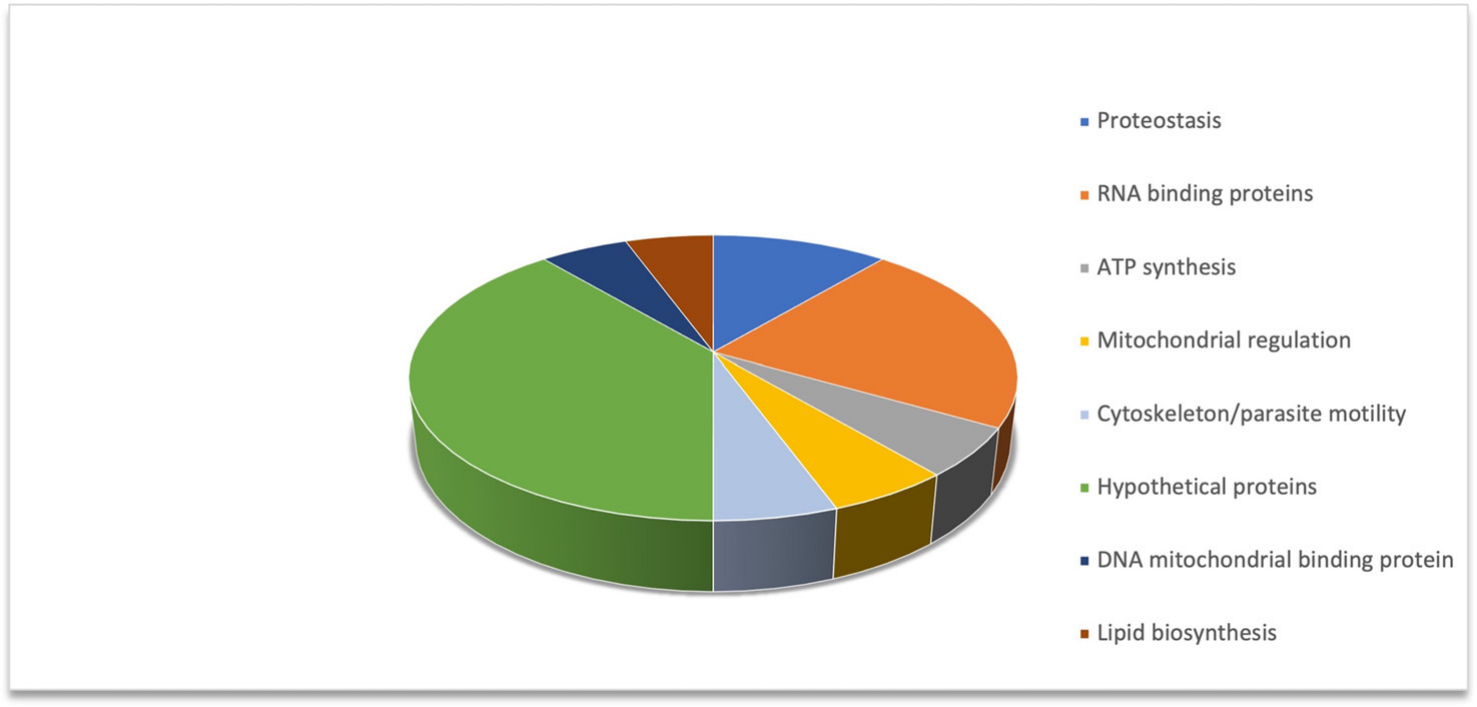
Functional classification of the 18 proteins specifically associated with the UTRs from the LYT1 mRNAs.

The 18 proteins specifically associated with the UTRs of LYT1 mRNAs were further analyzed looking for structural domains and/or functional annotations. Accordingly, these proteins were classified in the functional categories shown in Fig. 2, being hypothetical proteins (39%) and RBPs (22%) the largest categories.

## 2 Experimental Design, Materials and Methods

### 2.1 RNA baits

#### 2.1.1 PCR to obtain template DNA for *in vitro* transcription

The UTRs from the LYT1 mRNAs were amplified by reverse transcription (RT-PCR) using the oligonucleotides listed in the Supplementary Table S5 and cloned into the pGEM-Teasy plasmid (Promega, Inc., Madison, WI, USA) following standard procedures [10]. To obtain the DNA template for *in vitro* transcription, specific oligonucleotides with the T7 promoter sequence included at the 5′ end were designed as forward oligonucleotides for each UTR; all these oligonucleotides together with their reverse primers are listed in the Supplementary Table S6. Twelve ng per µl of DNA were used in PCR assays in a 30 *µ*L volume with buffer (Tris–HCl pH 9.0, KCl 50 mM, Triton X-100 0.1%), MgCl_2_ (2 mM), dNTPs mixture (0.4 mM each), oligonucleotides (0.4–0.8 µM), betaine (0.5 M), and high fidelity Taq polymerase enzyme 0.06 units per *µ*L (Roche, Inc., Mannheim, Germany). PCR reactions were performed on a C1000 thermal cycler (Biorad Laboratories In., Richmond, CA, USA), with the following amplification profile: initial denaturation at 95 °C for 3 min, 39 cycles at 92 °C for 30 s, banding temperature set according to the Tm of each oligonucleotide which in general was used in the range of 44–68 °C for 30 s, extension at 72 °C for 30 s and a final extension at 72 °C for 10 min.

#### 2.1.2 Preparation of RNA baits

The RNA baits were obtained using the commercial MEGAscript® *High Yield Transcription Kit* (Ambion Inc., Austin, TX, USA). In brief, for a final volume of 20 µL, 1 µg of purified DNA from each UTR was mixed in RNA polymerase enzyme buffer with 8 µL of rNTPs mixture (75 mM) and 2 µL of RNA polymerase enzyme (20 U/*µ*L). The mixture was incubated at 37 °C overnight. Afterwards, the DNA template was removed by incubation with DNase, and the UTRs baits were purified by the TRIzol® method (*Invitro*gen, Carlsbad, CA, USA). The *in vitro* transcription products were visualized by 1.5% agarose gel electrophoresis with MOPS buffer (0.4 M MOPS sodium salt, 150 mM sodium acetate, 10 mM EDTA, pH 7.5) and 6% formaldehyde.

### 2.2 *T. cruzi* cultures and protein extract preparation

*T. cruzi* epimastigotes (strain 058PUJ: DTU I) [11] were grown at 26 °C in LIT medium supplemented with 10% fetal bovine serum (FBS), penicillin 100 U/mL and streptomycin 100 *µ*g/mL. Trypomastigotes from the same strain of *T. cruzi* were purified from *T. cruzi*-infected green monkey kidney fibroblast cells (Vero cells; ATCC CCL-81, Manassas, VA). Infected cells were maintained in Dulbecco´s Modified Eagle´s medium (DMEM; Eurobio Inc, Les Ulis, France) supplemented with 10% FBS, 2 mM l-glutamine, 100 U/mL penicillin, 100 *µ*g/mL streptomycin and 0.01 M HEPES (Eurobio Inc, Les Ulis, France) at 37 °C with 5% CO_2_ [12]. *T. cruzi* parasites at the epimastigote stage were harvested when the culture density reached the logarithmic phase of growth (1 × 10^8^ cells/mL). Meanwhile, Vero cell-derived trypomastigotes were harvested in DMEM medium at a density of 1 × 10^7^ parasites per mL.

### 2.3 Pull-down

For pull-down protein capture assays, 1.3 mL of epimastigote or trypomastigote total protein extracts (1.5 mg/mL) were used for each assay; three biological replicates were performed. A 3′-biotinylated oligonucleotide (TcSLr/c3′biotinylated:5′-ATCAATAATATAGCGTTAGTTCCC-Biot-3′) complementary to the Spliced Leader (SL) sequence was used to capture the proteins interacting with the 5′UTRs (baits). Similarly, to capture proteins interacting with the 3′UTRs, a 5′-biotinylated poly-T oligonucleotide (biotinylated TcdT25:5′-Bio-T_25_–3′) was used. Then, streptavidin-coated magnetic beads (Promega, Inc., Madison, WI, USA) were incubated with the respective RNA bait previously hybridized to the biotinylated primer at 80 °C for 10 min. In brief, a 100 µL-mixture containing around 0.35 µg/µL of *in vitro* transcribed-RNA, 100 pM biotinylated oligonucleotide in 0.5 x SSC (75 mM NaCl, 7.5 mM Na_3_C_6_H_5_O_7_) was incubated with 0.25 mg of streptavidin-coated beads at room temperature (RT) for 15 min by gentle mixing by inversion. Next, the unbound RNAs was removed using a magnetic rack. The beads were blocked by incubation with 500 µL of binding buffer (NaH_2_PO_4_ 3.25 mM pH 7.4, NaCl 70 mM, 0.01% tween 20), and mixed with 0.4 M biotin and 0.4 nmoles of biotinylated primer in order to ensure the blocking of the streptavidin-coated magnetic beads (Promega, Inc., Madison, WI, USA). The supernatant was removed, and the beads were washed with 1 mL of binding buffer. Subsequently, 1.3 mL of the protein mixture (above) in binding buffer (containing RNAase Inhibitor 0.20 U/mL, yeast tRNA 0.13 mg/mL (*Invitro*gen, Carlsbad, CA, USA), *E. coli* total RNA 0.02 mg/mL and protease inhibitor 1 mg/mL) were added to each RNA bait (bound to magnetic beads, above). The mixture was incubated at RT with constant shaking for 20 min in a compact Lab Roller rotator carousel (Labnet International, Inc., Edison, NJ, USA). The unbound proteins were removed, and the beads were washed twice with 1 mL of wash buffer 1 (Tris–HCl 10 mm (pH 7.5), KCl 100 mM, MgCl_2_ 0.5 mM), once with 1 mL of wash buffer 2 (Tris–HCl 10 mM (pH 7, 5), supplemented with 0.5% CHAPS) and finally, washed three times with 1 mL of wash buffer 3 (Urea 200 mM, Thiourea 50 mM, CHAPS 0.25%, CO_3_K_2_ 5 mM).

Finally, the magnetic beads with the proteins bound to the RNA baits were subjected to 3 washes with 50 mM ammonium bicarbonate (1 mL). A 100 µL aliquot of the sample was mixed with 50 µL of 2x laemmli buffer (100 mM Tris–HCl (pH 6.8), 20% glycerol, 4% SDS, 0.02% bromophenol blue and 4% β-mercaptoethanol) and incubated at 95 °C for 10 min, and then analyzed by SDS-PAGE gel electrophoresis. Proteins were visualized by Coomassie G-250 colloidal staining (Biorad Laboratories In, Richmond, CA, USA). Afterwards, the proteins bound to the magnetic beads from the remaining sample (900 µL) were collected using a magnetic rack and allowed to dry at RT for 10 min and then stored at −80 °C until the LC-MS analysis. Three biological replicates were made for each RNA bait (UTR), including the *L. braziliensis* rRNA control. Samples were analyzed by LC/MS at the proteomics service of the CHÚ de Québec - centre de Recherche de l'Université Laval (http://www.crchudequebec.ulaval.ca/).

### 2.4 LC/MS proteomic analyzes

#### 2.4.1 Mass spectrometry

The on-beads digest and mass spectrometry experiments were performed by the Proteomics platform of the CHÚ de Québec Research Center, Québec, Canada. Briefly, proteins on beads were washed 3 times with 50 mM ammonium bicarbonate buffer and digested with trypsin (1 µg) (Promega, Madison, WI, USA) during 5 h at 37 °C, then they were desalted on a stage tip (C18) and vacuum dried before MS injection.

Peptide samples were separated by LC-MS/MS on an Ekspert NanoLC425 (Eksigent) coupled to a 5600+ mass spectrometer (AB Sciex, Framingham, MA, USA) equipped with a nanoelectrospray ion source. Peptides were separated with a linear gradient from 5 to 35% solvent B (acetonitrile, 0.1% formic acid) in 35 min, at 300 nL/minute on a picofrit column (Reprosil 3 u, 120A C18, 15 cm x 0.075 mm internal diameter). Mass spectra were acquired using a data dependent acquisition mode using Analyst software version 1.7. Each full scan mass spectrum (400–1250 *m/z*) was followed by collision-induced dissociation of the twenty most intense ions. Dynamic exclusion was set for a period of 3 s and a tolerance of 100 ppm.

#### 2.4.2 Database searching

MGF peak list files were created using Protein Pilot version 5.0 software (Sciex), and the searched was performed using Mascot (Matrix Science, London, UK; version 2.5.1) [1] on the TAX_Trypanosoma cruzi_5693database (55,107 entries), assuming the digestion with trypsin. Mascot was searched with a fragment ion mass tolerance of 0.100 Da and a parent ion tolerance of 0.100 Da. Deamidation of asparagine and glutamine and oxidation of methionine were specified in Mascot as variable modifications.

#### 2.4.3 Criteria for protein identification

Scaffold (version Scaffold_4.8.5, Proteome Software Inc., Portland, OR) was used to validate MS/MS based peptide and protein identifications. Peptide identifications were accepted if they could be established at greater than 95% probability by the Scaffold Local FDR algorithm. Protein identifications were accepted if they could be established at greater than 95% probability and contained at least 2 identified peptides. Protein probabilities were assigned by the Protein Prophet algorithm [13]. Proteins that contained similar peptides and could not be differentiated based on MS/MS analysis alone were grouped to satisfy the principles of parsimony.

### 2.5 Selection parameters and data

Selection criteria were established for the proteins identified by the Scaffold program (version Scaffold_4.8.5, Proteome Software Inc. Portland, OR, USA) having the following parameters: peptide reliability 99%, protein reliability 99%, false discovery rate (FDR) 0.01% and minimum number of 2 peptides per protein.

### 2.6 Functional classification proteins bound to the LYT1 UTRs

Based on the protein groupings generated with the Scaffold program (version Scaffold_4.8.5, Proteome Software Inc., Portland, OR), all differential binding and common binding proteins to the RNA baits (5′UTR mLYT1, 5′UTR kLYT1, and 3′UTR I, and 3′UTR II of LYT1), were analyzed to establish their functional classification as recorded in Fig. 2.

In the first instance, a protein blast (blastp) was performed on the following databases: GenBank (https://blast.ncbi.nlm.nih.gov), using the PSI-Blast algorithm (Position- Specific Iterated BLAST), Uniprot, (https://www.uniprot.org/; E-Threshold 0.001), Protein Data Bank (https://www.rcsb.org/; E-value Cutoff 0.001), and TritrypDB (https://tritrypdb.org; Expectation value 0.001) to establish percentages of identity against, the reference curated strain of *T. cruzi*. Also, complementary information for proteins was obtained from scientific literature.

Next, the sequence, and structure analysis of each protein was carried out with the following programs: InterproScan (https://www.ebi.ac.uk/), Phyre2 (http://www.sbg.bio.ic.ac.uk/) and Modeller (https://salilab.org/modeller/); in some cases, since there was no information on the protein structure, it was determined using the Robetta (The Baker lab, Seatle, WA, USA; http://robetta.bakerlab.org) and I-TASSER (Iterative Threading ASSEmbly Refinement; https://zhanggroup.org/I-TASSER/) programs. Specifically, Tc964 protein model coordinates were constructed using the SWISS-MODEL server (http://swissmodel.expasy.org) and model geometries were optimized by energy minimization with Deep View (version 4.1, Swiss-PdbViewer, Lausanne, Switzerland; http://spdbv.vital-it.ch/).

The Uniprot (https://www.uniprot.org/), Protein Data Bank (https://www.rcsb.org/), Protparam (https://web.expasy.org/), and Panther (http://www.pantherdb.org/) databases, were used to search for conserved structural domains.

## Ethics Statement

The protein dataset recorded in this manuscript did not involve the use of human beings or animal experiments; they are related to the research project "Characterization of protein factors associated with the regulation of the LYT1 protein of *Trypanosoma cruzi*" authorized by the Research and Ethics committee of the Facultad de Ciencias of Pontificia Universidad Javeriana (Project code 569–2012).

## CRediT authorship contribution statement

**Elizabeth Ruiz Márvez:** Investigation, Visualization, Data curation, Writing – original draft. **César Augusto Ramírez Segura:** Investigation, Conceptualization, Methodology. **José María Requena:** Writing – review & editing, Supervision. **Concepción J. Puerta:** Conceptualization, Methodology, Validation, Project administration, Funding acquisition, Writing – review & editing.

## Declaration of Competing Interest

The authors declare no conflict of interest. The authors declare that they have no known competing financial interests or personal relationships which have or could be perceived to have influenced the work reported in this article.

## Data Availability

Trypanosoma cruzi proteins identified in pull-down assays aimed to characterize RNA proteins interacting with LYT1 mRNAs (Original data) (Center for Computational Mass Spectrometry. Mass Spectrometry Interactive Virtual Environment MassIVE.)

## Appendix Supplementary Materials

Image, application 1

Image, application 2

Image, application 3

Image, application 4

Image, application 5
