# Supplementary material for: A dataset of proteins associated with Trypanosoma cruzi LYT1 mRNAs
Source: Data Brief. 2022 Feb 15;41:107953. doi: 10.1016/j.dib.2022.107953 (PMC8867043; doi:10.1016/j.dib.2022.107953)
Supplement: Supplementary file 5 [file mmc5.docx]

**Supplementary Materials**

**Supplementary tables:**

**Table S1.** Identified proteins in *T. cruzi* epimastigotes by their binding to the 5'UTR baits mLYT1, kLYT1 and rRNA *L. braziliensis* in first replicate (samples report for 22038–22040), second replicate (samples report for 22234–22236) and third replicate (samples report for 22237–22239). Also, the proteins associated with the 3'UTR baits I, II and rRNA *L. braziliensis* in first replicate (samples report for 22665,22666,23780,23781), second replicate (samples report for 22667,22668,23782,23783) and third replicate (samples report for 22669,22670,23784,23785).

**Table S2.** Identified proteins from *T. cruzi* trypomastigotes by their interaction with the 5'UTR mLYT1, kLYT1 and rRNA *L. braziliensis* baits in first replicate (samples report for 23762–23764), second replicate (samples report for 23765–23767) and third replicate (samples report for 23768–23770). Also, proteins interacting with 3'UTR I, II and rRNA *L. braziliensis* baits in first replicate (samples report for 23771–23773), second replicate (samples report for 23774–23776) and third replicate (samples report for 23777–23779).

**Table S3.** Proteins identified in at least two replicates to the LYT1 mRNAs UTRs used as baits in the epimastigote stage.

**Table S4.** Proteins identified in at least two replicates to the LYT1 mRNAs UTRs used as baits in the trypomastigote stage.

**Table S5.** Oligonucleotides used to amplify the UTRs of LYT1

**Table S6.** Oligonucleotides used to obtain the template DNA for *in vitro* transcription

**Table S5.** Oligonucleotides used to amplify the UTRs of LYT1

| Region | Oligonucleotides |
| --- | --- |
| 5´ UTR mLYT1 | **TC-SL-F:**  5´-AACTAACGCTATTATTGATACAGTT-3´ |
|  | **LYT1 4-r UJ:**  5´-GCATGAGAGCGAGCACGGC-3' |
| 5´ UTR kLYT1 | **TC-SL-F:**  5´AACTAACGCTATTATTGATACAGTT-3´ |
|  | **LYT188r:**  5´-CCATAAATTTATTGGCAATGGCAG-3´ |
| 3´ UTR LYT1 | **LY1648F:**  5-´CAGCTTGAAAACATGCTGG-3´ |
|  | **PoliT-*Eco*R1:**  5´CGGAATTCTTTTTTTTTTTTTTTTTTT-3´ |

**Table S6.** Oligonucleotides used to obtain template DNA for *in vitro* transcription

| Region | Oligonucleotides |
| --- | --- |
| Clone rRNA  *L. braziliensis* | **TcT7SL:**  5´***TAATACGACTCACTATAGGG***AACTAACGCTATTATTGAT-3´ |
|  | **ARNrPTr:**  5´-TTTTTTTTTTTTTTTTTTTTTTTTTCAATCACTAATTGGGCT-3´ |
| Clone 5´ UTR mLYT1 | **TcT7SL:**  5´-***TAATACGACTCACTATAGGG***AACTAACGCTATTATTGAT-3´ |
|  | **LYT1 4-r UJ:**  5´-GCATGAGAGCGAGCACGGC-3' |
| Clone 5´ UTR kLYT1 | **TcT7SL:**  5´-***TAATACGACTCACTATAGGG***AACTAACGCTATTATTGAT-3´ |
|  | **LYT188r:**  5´-CCATAAATTTATTGGCAATGGCAG-3´ |
| Clone 3´ UTR-I /  Clone 3´ UTR-II LYT1 | **TcLY3UT7F:**  5´-***TAATACGACTCACTATAGGG***CTGGCAGCTGATTGA-3´ |
|  | **PoliTd:**  5´-CGGAATTCTTTTTTTTTTTTTTTTTT-3´ |

**Supplementary figures:**

**
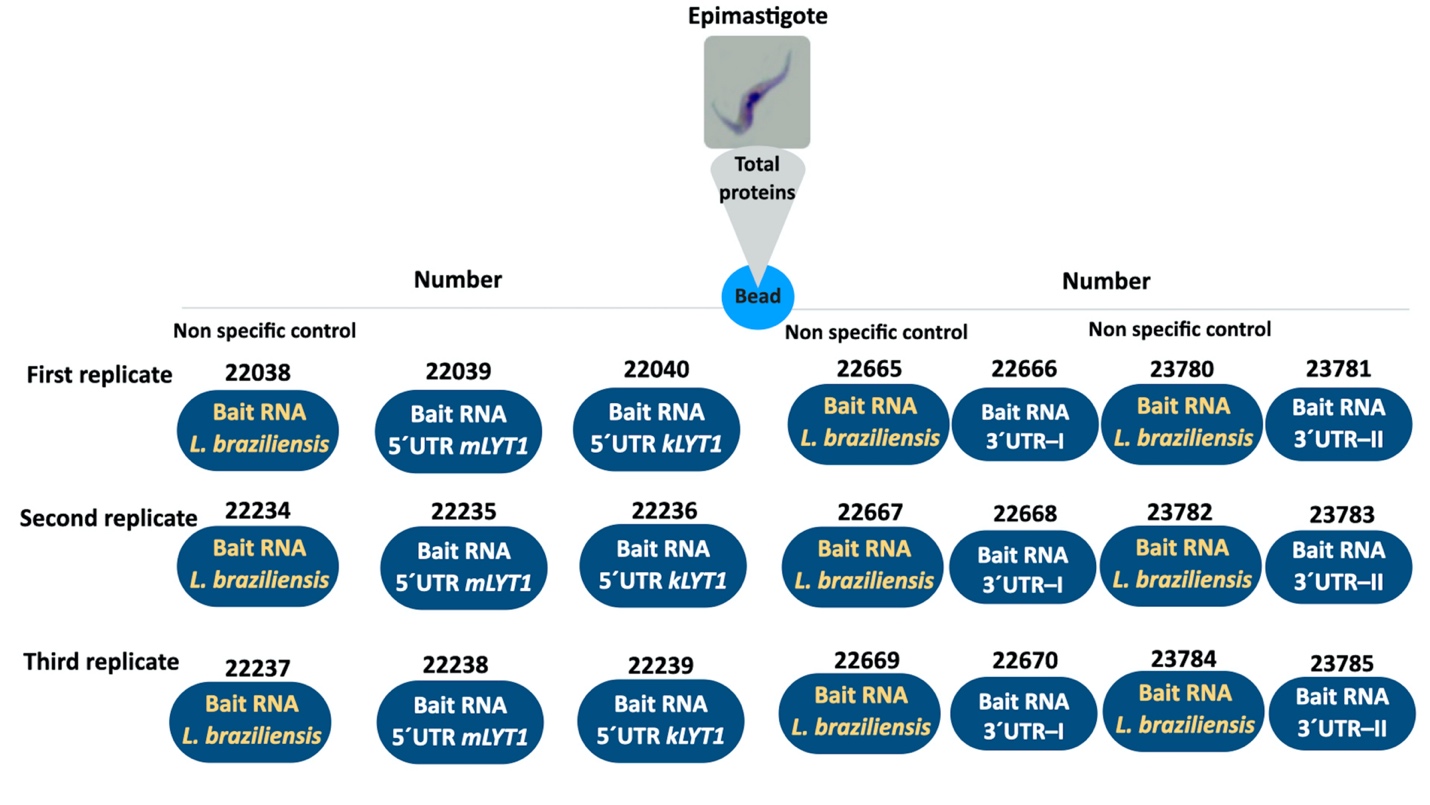
**

**Fig. S1.** Protein samples bound to specific RNA bait (5'UTR mLYT1, 5'UTR kLYT1, 3'UTR-I, 3'UTR-II) and identified by LC/MS using *L. braziliensis* rRNA as a non-specific control for each biological replicate on the epimastigote stage. The number refers to the number given for the analysis of the samples at the CHÚ de Québec proteomics Service-Laval University Research Center (Québec, Canada), recorded in turn in the supplementary data.

**
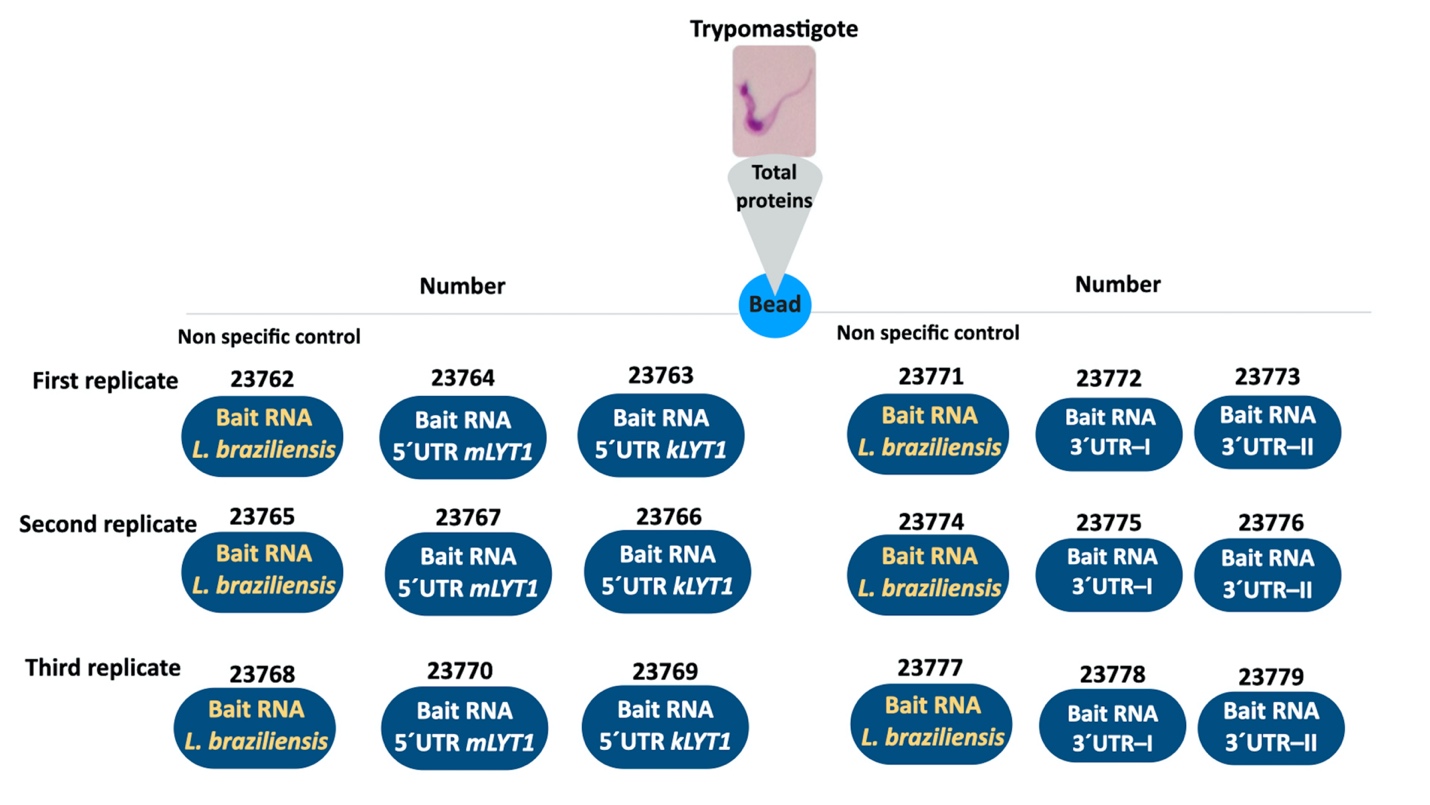
**

**Fig. S2.** Protein samples bound to specific RNA bait (5'UTR mLYT1, 5'UTR kLYT1, 3'UTR-I, 3'UTR-II) and identified by (LC/MS) using *L. braziliensis* rRNA as a non-specificity control for each biological replicate on the trypomastigote stage. The number refers to the number given for the analysis of the samples at the CHÚ de Québec proteomics Service-Laval University Research Center (Québec, Canada), recorded in turn in the supplementary data.
